# Supplementary material for: ChatGPT as a Tool for Biostatisticians: A Tutorial on Applications, Opportunities, and Limitations
Source: Stat Med. 2025 Oct 23;44(23-24):e70263. doi: 10.1002/sim.70263 (PMC12548020; doi:10.1002/sim.70263)
Supplement: Supplementary file 1 — Data S1. Supporting Information. [file SIM-44-0-s001.zip › Supplementary Materials Additional.zip!/3.4_Sample_Sizes_Planning_for_Survival_Outcomes/3.4_Sample_Sizes_Newer_Versions/03_chat_sample_size_o3.pdf]

OpenAI

03_chat_sample_size_o3

please clear memory

I don’t retain a permanent memory of our past conversations—each session is temporary, and once it ends I no longer have access to the details. If you’d like to start fresh right now, just let me know and I’ll respond as though this is a brand-new chat.

You take the role of an expert biostatistician in this chat.

Absolutely—happy to put on my biostatistician hat. How can I help you today?

I would like to compare a new therapy with the standard therapy regarding survival in advanced pancreatic carcinoma. Please perform a sample size calculation and incorporate the following parameters:
o Primary outcome: Overall survival.
o Median survival time in the standard therapy group: 12 months.
o Median survival time in the new therapy group: 18 months.
o Power = 80% (1 - β = 0.8)
o a two-sided significance level of α = 0.05.
o The allocation ratio should be 1:1 (equal group sizes).
o Patients will be followed up for 2 years.
o We expect a dropout rate of 15%.
Use the data analysis mode
-

Survival Sample Size Summary

1	Target power	0.8	
2	Two-sided α	0.05	
3	Median OS (standard)	12 months	
4	Median OS (new)	18 months	
5	Hazard ratio (new vs standard)	0.667	
	Quantity	Value	

Key results

- **Hazard ratio (new ÷ standard): 0.67** (derived from the median OS values 18 mo vs 12 mo).
- **Events required (log-rank/Schoenfeld)**: **191 deaths**.
- **Total sample size (no dropout)**: **283 patients** → **142 per arm**.
- **Total sample size after adding 15 % dropout cushion**: **333 patients** → **167 per arm**.

The calculation assumes:

- Exponential survival distributions.
- All patients begin follow-up immediately and are observed for a fixed **24 months**.
- Equal 1:1 allocation, two-sided α = 0.05 and 80 % power.

The interactive table above shows the full derivation and intermediate values so you can adjust any assumptions as needed. Let me know if you'd like to explore different follow-up lengths, accrual periods, power, or dropout scenarios!
